# Mitochondrial Metabolomics in Cancer: Mass Spectrometry-Based Approaches for Metabolic Rewiring Analysis and Therapeutic Discovery

**DOI:** 10.3390/metabo15080513

**Published:** 2025-07-31

**Authors:** Yuqing Gao, Zhirou Xiong, Xinyi Wei

**Affiliations:** 1 School of Pharmacy and Medical Technology, Putian University, Putian 351100, China; 2 Fujian Province University, Key Laboratory of Pharmaceutical Analysis and Laboratory Medicine (Putian University), Putian 351100, China; 3 Fujian Province University, Key Laboratory of Medical Microecology (Putian University), Putian 351100, China; 4 State Key Laboratory of Drug Research, Shanghai Institute of Materia Medica, Chinese Academy of Sciences, Shanghai 100864, China; 5 University of Chinese Academy of Sciences, Beijing 100049, China; 6 School of Nursing, Putian University, Putian 351100, China

**Keywords:** mitochondrial metabolomics, mass spectrometry, mitochondria isolation, targeted metabolomics, untargeted metabolomics

## Abstract

Mitochondria, pivotal organelles in cellular metabolism and energy production, have emerged as critical players in the pathogenesis of cancer. This review outlines the progress in mitochondrial profiling through mass spectrometry-based metabolomics and its applications in cancer research. We provide unprecedented insights into the mitochondrial metabolic rewiring that fuels tumorigenesis, metastasis, and therapeutic resistance. The purpose of this review is to provide a comprehensive guide for the implementation of mitochondrial metabolomics, integrating advanced methodologies—including isolation, detection, and data integration—with insights into cancer-specific metabolic rewiring. We first summarize current methodologies for mitochondrial sample collection and pretreatment. Furthermore, we then discuss the recent advancements in mass spectrometry-based methodologies that facilitate the detailed profiling of mitochondrial metabolites, unveiling significant metabolic reprogramming associated with tumorigenesis. We emphasize how recent technological advancements have addressed longstanding challenges in the field and explore the role of mitochondrial metabolism-driven cancer development and progression for novel drug discovery and translational research applications in cancer. Collectively, this review delineates emerging opportunities for therapeutic discovery and aims to establish a foundation for future investigations into the therapeutic modulation of mitochondrial pathways in cancer, thereby paving the way for innovative diagnostic and therapeutic approaches targeting mitochondrial pathways.

## 1. Introduction

Mitochondria, the essential metabolic hubs [1], have been increasingly regarded as critical for human health and disease [2]. Mitochondria are semi-autonomous organelles possessing their own DNA, capable of replication, independent transcription and translation, and they contain four functional compartments partitioned by the phospholipid bilayer: the outer membrane (OM), intermembrane space (IMS), inner membrane (IM), and matrix [3,4]. The IM houses various mitochondrial permeability transition pores (MPTPs), permeable to solutes and small molecules (molecular weight up to 1500 Da), which are vital for regulating cell signaling and even cell fate [5]. The mitochondrial electron transport chain (ETC) and supercomplexes are also localized at the IM, which is a critical site of ATP production and various metabolic processes [6]. The ETC is composed of five complexes: complex I (NADH dehydrogenase), complex II (succinate dehydrogenase), complex III (cytochrome [1] c reductase), complex IV (cytochrome c oxidase), and complex V (ATP synthase). The ETC consists of 76 protein subunits [7], of which 13 are encoded by mitochondrial DNA (mtDNA) [8]. The mtDNA, as an independent mitochondrial genome, regulates mitochondrial transcription and replication together with other transcription factors, which are all core components for mitochondrial structure and function [9]. The space between the outer and inner membranes is the IMS, playing key roles in protein transport, folding, and assembly, along with oxidative phosphorylation (OXPHOS) [10]. Additionally, the IM can increase the ATP-producing surface area by folding into cristae. The matrix, a dense solution filled within the IM, contains diverse metabolic enzymes and substrates involved in the citric acid cycle, glycolysis, one-carbon metabolism, and fatty acid synthesis and beta-oxidation, along with glutamine metabolism [11,12,13], participating in the majority of biochemical reactions.

Mitochondria often emerge as cellular powerhouses. However, increasing evidence indicates that mitochondria are not only energy centers but also vital hubs for cellular metabolism, stress responses, inflammation, signal transduction, and cell fate [1,14]. In recent decades, dysfunction of core mitochondrial targets has been closely linked to over 150 distinct diseases, ensuring that mitochondrial proteins are highly relevant therapeutic targets [15]. However, the functions of about 40% of mitochondrial targets still remain unknown. Moreover, the mechanisms underlying these mitochondrial target-related diseases, severely hindering diagnosis and drug development, also urgently need elucidation [16].

Small-molecule metabolites (with molecular weights between 50 and 1500 Da) are the ultimate products of biological processes and direct manifestations of functional changes [17]. Metabolomics, emerging after genomics and proteomics, is a novel omics technique that combines pattern recognition methods and bioinformatics tools to enable the high-throughput detection of complete sets of metabolites (metabolome) and to track their alterations in different biological systems [18]. Metabolomics involves the unbiased qualitative or quantitative analysis of the whole metabolome in a biological sample under a specific status to identify differential metabolites between different conditions, facilitating early disease diagnosis, drug target discovery, disease mechanism research, and drug development [19]. Currently, the estimated size of the human metabolome ranges from 8000 to 114,100 metabolites, depending on different analytical methodologies [20], containing mitochondrial core metabolites such as intermediates of the tricarboxylic acid (TCA) cycle, glycolysis products, amino acids and their derivatives, fatty acids and their oxidation-related metabolites, ketone bodies, and nucleotides, along with coenzymes (especially coenzyme A and its derivatives) [21,22]. Given that metabolomics could depict panoramic views of the mitochondrial metabolic status in multiple samples, it has been widely utilized in mitochondrial research.

Although metabolomics is a powerful tool for the clear delineation of metabolic changes, some challenges still persist. Firstly, obtaining highly pure mitochondria (subpopulations) is difficult due to the lack of effective methods, which might induce contaminations from other membrane-bound organelles [19,23,24]. Secondly, the complex and time-consuming procedure of mitochondria isolation could lead to alterations of mitochondrial metabolic states. It is hard to control mitochondrial quality even when keeping the temperature low during the whole isolation process [25]. Moreover, reagents used in mitochondrial isolation contain inorganic ions that can interfere with mass spectrometry signals [26]. Lastly, compared to other biological samples, mitochondrial samples are difficult to obtain, leading to the low abundance of mitochondrial metabolomes, which require accurate, highly sensitive, and broad-range detection methods [27].

In light of difficulties and developments, this review provides a comprehensive guide for the implementation of mitochondrial metabolomics, integrating advanced methodologies—including isolation, detection, and data integration—with insights into cancer-specific metabolic rewiring. We emphasize how recent technological advancements have addressed longstanding challenges in the field and delineate emerging opportunities for therapeutic discovery.

## 2. Collection and Storage of Mitochondrial Samples

The collection and storage procedures are of great importance; they determine the metabolic profiles of the mitochondria. Mitochondria, fueling numerous indispensable biochemical reactions and cellular processes, are susceptible to environmental changes and relatively low abundance in the whole cell, which requires ice-cold isolation and further enrichment [28]. Herein, we outlined the current practices of processing mitochondrial samples for metabolomics study, including sample collection, quantity and quality control, and storage, along with extraction.

### 2.1. Collection

**Figure 1:**
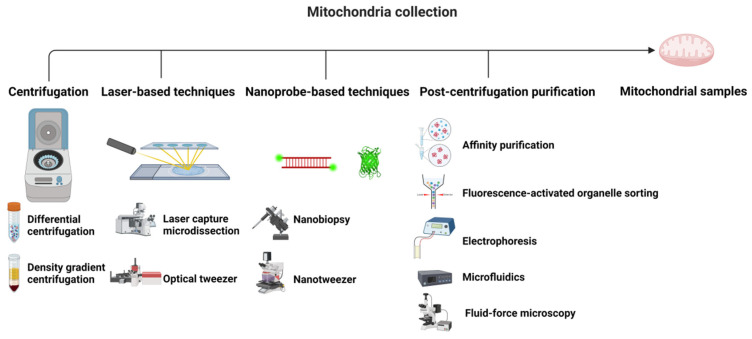
Overview of mitochondrial collection techniques. This diagram outlines key methods for mitochondrial collection. Conventional centrifugation (differential/density gradient) leverages sedimentation for large-scale separation but is slow and yields moderate purity. Laser-based methods (capture microdissection, optical tweezers) enable single-cell/subcellular precision for heterogeneity studies, though they risk laser-induced damage. Nanoprobe approaches (nano-biopsy, optical tweezers) combine microfluidics and electroporation for non-invasive, real-time tracking but demand advanced expertise. Post-centrifugation purification (affinity, fluorescence-activated sorting, electrophoresis, microfluidics, fluidic microscopy) further refines mitochondrial subpopulations via antibodies, fluorescent markers, or physical properties, supporting high-resolution metabolomics and high-throughput analyses. Collectively, these complementary strategies balance yield, purity, and resolution to advance our understanding of mitochondrial function and heterogeneity.

Collection is the first but most important step for mitochondrial isolation, which is mainly achieved through manual homogenization [25]. Centrifugation, especially differential or density gradient centrifugation (DC or DGC), typically the next step after homogenization, is the most classic and widely accepted approach to obtain large-scale cellular mitochondrial fractions. However, centrifugation is considerably time and resource consuming, and it is also far from isolating distinct mitochondrial subpopulations [29]. To study the heterogeneity of mitochondria, laser-capture microdissection (LCM) and nanoprobe-based techniques are applied to nanoscale mitochondria isolation. Moreover, post-centrifugation purification methods, including affinity purification (AP), fluorescence activated organelle sorting (FAOS), electrophoresis, microfluidics, and fluid-force microscopy (FluidFM) were sequentially combined with centrifugation for more purified mitochondrial subpopulations (as shown in Table 1 and Figure 1).

#### 2.1.1. Centrifugation

According to different mass and sedimentation properties, differential and density gradient centrifugation (DC and DGC) are employed for the separation of organelles, such as mitochondria [30]. In DC, a cell or tissue suspension is centrifuged at increasing speeds, whereas, in DGC, it is spun through a gradient of mediums with gradually increasing density [28,31]. DGC offers a more purified mitochondrial fraction and results in a lower quantity than DC [32]. Mitochondrial output and quality can be enhanced by incorporating detergents and moderating the homogenization and centrifugation rates [33]. Both DC and DGC are reliable and effective for obtaining functional mitochondria, though they demand substantial resources and time. Depending on the specific requirements of the study, DC might be the preferred choice for achieving higher yields at the cost of lower purity [30]. Centrifugation has become the dominant approach for large-scale mitochondrial isolation, but a combination of post-centrifugation methods with centrifugation is gaining more popularity.

#### 2.1.2. Laser-Based Techniques

Laser capture microdissection (LCM) and optical tweezers (OTs) are excellent tools using laser beams for gathering cell populations, single cells, and subcellular organelles. Laser capture microdissection (LCM) is utilized for single-cell isolation by employing nanosecond pulses from a nitrogen laser to separate a delineated cell. Moreover, gravity assistance or propulsion induced by a laser pulse could accelerate translocation and capture, guiding the target into the collection vessel [34]. It has been reported that LCM could be applied to isolate mitochondria at the single-cell or subcellular level, and even to investigate the clonal expansion of mtDNA mutations [35,36]. Optical tweezers (OTs), able to simultaneously lyse cells, could accurately trap organelles into a specific laser beam [37]. LCM and OTs are exciting techniques that are gold standard methods for studying mitochondrial diversity at the single-cell level [38]. However, when attempting to repeatedly isolate mitochondria from tiny subcellular targets, the use of LCM and OTs is constrained due to their damage inflicted on mitochondria and mtDNA by laser induction [39]. Apart from flow cytometry, LCM and OT are gold standard methods for studying mitochondrial diversity at the single-cell level [40].

#### 2.1.3. Nanoprobe-Based Techniques

Advances in nanotechnology have shown remarkable potential for the precise isolation of mitochondria [41,42]. Micromanipulators equipped with micropipettes are capable of successful isolation and transplantation of nuclei [43]. Moreover, nanoprobes permit a more precise and non-invasive manipulation of biomolecules and organelles than those larger microscale tools [44]. However, there exists an obstacle to isolating mitochondrial samples using this technology. More evidence indicates that nanoprobes could allow for the longitudinal tracking of mitochondria and comparison of mitochondrial heterogeneity across and within foci deficiency, which minimize disruptions to cell viability and their intracellular contents [45,46]. Two key methods for the nanomanipulation of mitochondria have been developed: nanobiopsy, where mitochondria are isolated from living cells through electrowetting within a nanopipette; and nanotweezers (NTs), where cells are cultured on a matrix of channels, and a transient electric pulse induces transient membrane pores allowing precise mitochondrial extraction [47]. Nanoprobe techniques address contamination and activity loss during isolation by enabling the non-invasive extraction of functional mitochondrial subpopulations directly from living cells—critical for preserving labile metabolites and in situ metabolic states.

#### 2.1.4. Post-Centrifugation Purification

Post-centrifugation purification is vital in mitochondrial metabolomics for enhancing sample purity and functionality, enabling more precise metabolic analysis. Techniques such as affinity purification (AP), fluorescence-activated organelle sorting (FAOS), electrophoresis, microfluidics, and fluid-force microscopy (FluidFM) have been employed to isolate mitochondria for preserving their biological activity, thereby facilitating the research of mitochondrial function.

Affinity purification (AP) effectively isolates mitochondria from crude fractions by leveraging specific interactions between mitochondrial surface proteins and antibody-coated magnetic beads [48]. AP not only ensures high yield and purity but also guarantees the separation of mitochondrial subpopulations [49,50], with small sample consumption and short processing time [48]. Despite the high reagent cost, AP still gradually becomes the primary choice for the isolation of large-scale mitochondria and specific subpopulations after centrifugation due to its high performance [50,51].

FAOS could select mitochondria from other cellular components or distinct mitochondria groups in specific droplets upon identifying fluorescent labels [52]. FAOS could separate a high quantity of functional and pure mitochondrial subsets, reducing the overall material demand, while FAOS is limited by the cytotoxicity of fluorescent labels such as fluorescent dyes. Although multifarious labels are increasing to replace those potentially toxic labels [53], fluorescent labels could still induce mitochondrial clumping and mtDNA copy number variation [54]. Nevertheless, FAOS is the most effective method for high-throughput and high-yield mitochondrial separation from large samples [55].

Capillary electrophoresis (CE), free-flow electrophoresis (FFE), and field-flow fractionation (FFF) are the three most common electrophoresis methods for separating mitochondria subsets. In CE and FFE, mitochondria are separated based on their isoelectric properties under an electric field, through the application of an electric field [56], while FFF sorts particles according to their size and mass via perpendicular cross flow. Research suggests that FFE is best suited for high-throughput studies, prioritizing mitochondrial purity over yield [57]. In contrast to FFE, CE is suitable when purity is preferred over a high yield (due to the retention of mitochondria in the narrow capillary tube), having a better signal-to-noise ratio than FAOS and exhibiting lower throughput but requiring a smaller initial sample [58]. Unlike FFE and CE, FFF is most effective when a rapid, high mitochondrial yield is needed, even at the cost of purity [59].

Microfluidics emerges as a highly effective method for isolating highly purified and functional mitochondrial components from limited initial samples [40,60,61]. Despite its lower yield and limited capacity to provide detailed information about mitochondrial subsets, microfluidics has become almost the most suitable method for effectively achieving highly purified and functional mitochondrial components from constrained samples [62,63].

FluidFM is a combination of microfluidics and an atomic force microscope (AFM), which could aspirate cellular components by applying negative pressure through the AFM tip with a controlled system [64]. FluidFM, much like nanoprobe-based techniques, is suitable for the isolation of specific subcellular mitochondria subpopulations with minimal effects on cell viability and precision of targeting specific subcellular locations, emphasizing high-resolution sampling over yield. FluidFM could isolate intact mitochondria independent of calcium, which would be transplanted and even fused into mitochondria from host cells [65].

**Table 1:** Methodologies for mitochondria collection.

Isolation Method	Timing	Yield	Purity	Advantages	Limitations	Refs.
DC	Moderate	High	Moderate	Reliable for large-scale isolation	Time-consuming and resource-intensive	[28,30,31]
DGC	Long	Low	High	Higher purity of mitochondrial fractions	Low yield; complex procedure	[28,30,32]
Laser-based techniques	Short	Variable	Variable	Effective for single-cell isolation	Potential damage to mitochondria and mtDNA	[34,35,36,37,38,39,40]
Nanoprobe-based techniques	Real-time	Variable	Variable	Precise and non-invasive	Challenges in sample isolation	[41,42,43,44,45,46,47]
AP	Short	High	High	High specificity and purity	Higher reagent costs	[48,49,50,51]
FAOS	Real-time	High	High	Effective for high-throughput applications	Cytotoxicity of fluorescent labels	[52,53,54,55]
CE	Variable	Low	High	Good signal-to-noise ratio	Lower throughput; requires smaller samples	[58]
FFE	Variable	High	Moderate	Rapid separation for high-throughput studies	Trade-off with purity	[57]
FFF	Variable	High	High	Rapid and effective	Trade-off with purity	[59]
Microfluidics	Variable	Low	High	Minimal damage to organelles	Limited information on mitochondrial subsets	[40,60,61,62,63]
FluidFM	Real-time	Variable	Variable	High-resolution sampling with minimal effects	Challenges in targeting specific locations	[64,65]

### 2.2. Quantity and Quality Control of Mitochondrial Samples

Given the variable abundance and unknown quality of mitochondrial samples after separation, the normalization of quantity and quality control is necessary. For quantification of isolated mitochondrial samples, total protein concentration and the relative mtDNA copy number are two primary evaluation criteria [66,67]. First, to assess the total mitochondrial protein samples, three approaches are available, including Bradford, Lowry, and bicinchoninic acid (BCA) assays. The Bradford assay is more suitable for high-concentration mitochondrial samples, while Lowry and BCA assays are recommended due to their high sensitivity [68]. Second, relative mtDNA copy numbers were often measured by SYBR green-based quantitative real-time PCR (qPCR) assays as reported previously [69].

Alongside the quantification of isolated mitochondrial samples, the quality of these samples regarding purity, activity, and membrane integrity of mitochondria needs further detection. The purity of mitochondrial samples could be examined in Western blot (WB) using markers of different subcellular fractions including mitochondria, nuclei, and cytoplasm [23,70]. A transmission electron microscope (TEM) is also applied to evaluate the purity and structure of mitochondrial isolates [19,71]. Vital standards for mitochondrial quality control, including measurements of OXPHOS and mitochondrial respiratory chain enzymatic activities, monitor mitochondrial activity [72]. Mitochondrial reactive oxygen species (mtROS) production is often considered the gold standard for the tracking of mitochondrial OXPHOS [73]. Moreover, mitochondrial respiratory chain enzymatic activities are often detected by the oxygen consumption rate (OCR) and extracellular acidification rate (ECAR) [74]. The membrane integrity of mitochondria is assessed via TEM, SYTOX staining, and MPTP assay [75,76].

### 2.3. Storage

The temperatures for mitochondria collection and storage are generally −80 °C, 0 °C, and 4 °C. The entire process of mitochondrial isolation is often performed at a temperature ranging from 0 °C to 4 °C. During mitochondria separation, the working solution is often pre-cooled and kept at 0 °C to 4 °C [29]. The entire centrifugation procedure is maintained at 4 °C [77]. Isolated mitochondria could retain their integrity when placed on ice for a duration of 5–6 h [78]. To ensure long-term stability, mitochondrial samples should be stored at a temperature not lower than −70 °C, with protein concentration above 40 mg/mL. Adding dimethyl sulfoxide or ethylene glycol and avoiding multiple freeze–thaw cycles could help for long-term preservation [79].

## 3. Mass Spectrometry-Based Analysis for Mitochondrial Metabolomics

Mitochondrial metabolome encompasses numerous key molecules involved in energy production and life activities: intermediates of the tricarboxylic acid (TCA) cycle (citrate, isocitrate, α-ketoglutarate, succinate, fumarate, malate, 2-hydroxyglutarate, and oxaloacetate, etc.), glycolysis products (pyruvate, lactate, 3-PGA, and G3P, etc.), amino acids and their derivatives (glutamine, aspartate, arginine, serine, glycine, proline, and methionine, etc.), fatty acids and their oxidation-related metabolites (acetate, propionate, and butyrate, etc.), ketone bodies (β-hydroxybutyric acid and acetyl-acetate, etc.), and nucleotides (ATP, ADP, AMP, GTP, GDP, GMP, NAD^+^/NADH, NADP^+^/NADPH, FAD, and FADH2), along with coenzymes (especially coenzyme A and its derivatives, including acetyl-CoA, malonyl-CoA, succinyl-CoA, acylcarnitines, and GSH/GSSG, etc.) [80,81], etc. Mitochondrial metabolomics faces analytical challenges due to metabolite compartmentalization and diverse properties.

To address these challenges, mass spectrometry (MS)-based platforms have been optimized with methodological adaptations to enhance spatial resolution and sensitivity. For polar metabolites, including tricarboxylic acid (TCA) cycle intermediates (α-ketoglutarate, succinate), acylcarnitines (C2–C18), redox pairs (NAD^+^/NADH, FADH_2_/FAD, and GSH/GSSG), hydrophilic interaction liquid chromatography–mass spectrometry (HILIC-MS) offers a robust approach [82]. Alternatively, gas chromatography–mass spectrometry (GC-MS), when coupled with appropriate derivatization techniques, offers a robust approach for analyzing thermally stable polar metabolites [83]. For analysis of fatty acids and membrane lipids, including cardiolipins (CL 72:8, CL 74:7) [84], lysophosphatidic acid (LPA) [85], and mitochondrial membrane fatty acids (C16:1, C18:2) [86], reversed-phase liquid chromatography–mass spectrometry is particularly effective. Moreover, matrix-assisted laser desorption/ionization time-of-flight mass spectrometry coupled with orbitrap mass analyzer (MALDI-TOF/Orbitrap) imaging provides a powerful tool for visualizing the spatial distribution of lipids within mitochondria [87,88]. To assess the metabolic flux of pyruvate, β-hydroxybutyrate (β-HB), glutamine, etc., stable isotope tracing coupled with liquid chromatography–high-resolution mass spectrometry (LC-HRMS) offers a powerful strategy [89,90].

### 3.1. Sample Pretreatment

The metabolites in mitochondria show huge differences in chemical structures, physicochemical properties, and concentration distribution ranges, which seriously hinder the subsequent detection and analysis. Therefore, different sample pretreatment methods are required for mitochondrial metabolomics.

GC-MS analysis is frequently applied to mitochondrial primary metabolites, such as citrate, α-ketoglutarate, malate, and amino acids, etc. [91]. Generally, for GC-MS detection, sample pretreatment is carried out in the sequence of extraction first and then derivatization. Mitochondrial extracts are prepared using ice-cold methanol/water (9:1 *v*/*v*) with 0.1% formic acid to quench enzymatic activity. After centrifugation (15,000× *g*, 10 min, 4 °C), supernatants are derivatized with 20 μL of methoxyamine hydrochloride (20 mg/mL in pyridine) at 30 °C for 90 min, followed by N-methyl-N-(trimethylsilyl)trifluoroacetamide (MSTFA) silylation for GC-MS compatibility [92]. As for derivatization, an alkylating reagent, methoxyamine hydrochloride in pyridine, which can derivatize a variety of compounds, including amides, amines, amino acids, sugars, hydroxy compounds, carboxylic acids, and inorganic anions, is often utilized [93].

The hydrophilic and highly polar mitochondrial metabolites, which are generated from the metabolism of the respiratory electron transport chain (including acylcarnitines, redox pairs, and ketones, etc.), are mainly detected by LC-MS, especially using hydrophilic interaction liquid chromatography (HILIC) and reversed-phase liquid chromatography (RPLC) systems [94,95]. For acylcarnitines and redox pairs, a dual-phase extraction (methanol/chloroform/water, 8:1:1 *v*/*v*) preserves labile species [96], with N-ethylmaleimide (NEM, 10 mM) added to stabilize thiol-containing metabolites (ketone bodies and redox pairs) [97,98].

For spatially resolved metabolic profiling within cellular compartments, mass spectrometry imaging (MSI), particularly matrix-assisted laser desorption/ionization (MALDI)-MSI, offers a powerful approach to map metabolite distributions. Analysis of mitochondrial lipids, including fatty acids and membrane phospholipids, necessitates specialized sample preparation compatible with MSI. This typically involves extraction using a chloroform/methanol (2:1, *v*/*v*) solvent system, followed by Folch partitioning to isolate the lipid fraction and effectively remove interfering salts. The purified lipid extract is then co-crystallized with an appropriate MALDI matrix directly onto the imaging target plate. Critical for ionization efficiency and spectral quality, matrix selection is tailored to the analyte polarity and desired ionization mode: 9-aminoacridine (9-AA) is predominantly employed for the negative-ion mode detection of acidic lipids such as phospholipids and sulfatides, whereas 2,5-dihydroxybenzoic acid (DHB) is favored for positive-ion mode analysis targeting neutral lipids including glycerolipids and sphingolipids [96].

Stable isotope-resolved metabolomics (SIRM) is indispensable for dissecting dynamic metabolic fluxes and pathway activities within mitochondria. Sample preparation for SIRM demands exceptionally rapid metabolic quenching to precisely preserve the in vivo isotopic labeling patterns at the time of sampling, coupled with high-yield extraction of targeted metabolite classes. This is frequently achieved through adaptations of the aforementioned extraction methodologies (e.g., ice-cold methanol/water or methanol/chloroform/water biphasic systems), with stringent optimization of quenching kinetics to minimize post-sampling metabolic activity and potential isotope exchange artifacts. Furthermore, stabilization strategies, such as the incorporation of N-ethylmaleimide (NEM) to alkylate and protect thiol groups in labeled redox pairs (e.g., glutathione disulfide/glutathione, GSSG/GSH), are essential to maintain the integrity of labile species. Subsequently, the isotopically enriched extracts are subjected to analysis via LC-MS or GC-MS platforms, where quantification of mass isotopomer distributions enables the reconstruction of metabolic flux networks [99].

### 3.2. Chromatography-Based Separation Techniques

The mitochondrial metabolome has an extremely complex composition. Appropriate chromatography-based separation methods are of great help in constructing sensitive and reproducible analytical methods.

GC is mainly applied to separate metabolites with small molecular weights (citrate, lactate, α-KG, 2-HG), high volatility (acetate, pyruvate), low electrospray ionization efficiency (folate, acetyl-CoA, NADPH), and medium-sized charge (sugar monophosphates) [83], whose volatility and detectability were enhanced through MSTFA derivatization [100]. A DB-5MS UI column (Agilent Technologie, Santa Clara, CA, USA) (30 m × 0.25 mm × 0.25 μm) with a programmed temperature gradient (80 °C to 320 °C) is recommended for the optimal separation of derivatized metabolites [101]. Electron ionization (EI) (Q Exactive MS, Thermo Fisher Scientific, Waltham, MA, USA) at 70 eV, coupled with selected ion monitoring (SIM) for characteristic fragment ions (e.g., citrate 273–113 *m*/*z*), enables differentiation of mitochondrial-derived metabolites from their cytosolic counterparts [101].

In contrast, LC is mostly utilized for the separation of hydrophilic metabolites, comprising nucleosides, amino acids, and monosaccharides [102]. Additionally, ion mobility spectrometry (IMS) can be used for the separation of isomers, involving leucine and isoleucine, as well as fructose and glucose, etc. [103]. HILIC-MS using ZIC-HILIC columns (EMD Millipore, Burlington, VT, USA) provides complementary separation for underivatized polar acids, leveraging mobile phases of acetonitrile/water with ammonium formate buffers (pH 3.4–6.8) and electron spray ionization (ESI) in negative mode for enhanced sensitivity [104]. HILIC-MS with dansyl chloride derivatization also enables the sensitive quantification of redox pairs and ketones [105]. Amino acids (glutamine, proline) and nucleotides (ATP, NAD^+^) are profiled using AccQ-Tag™ pre-column (Waters Corporation, Milford, CT, USA) derivatization followed by HILIC-MS/MS or ion-pairing LC-MS/MS with tributylamine additives [106] (as shown in Table 2). These methods resolve isobaric species (e.g., leucine/isoleucine) and quantify labile metabolites like acetyl-CoA, integrating seamlessly with stable isotope tracing for flux analysis [107,108]. RP-LC-MS using C18 column (2.1 × 100 mm, 1.8 μm) (Waters Corporation, Milford, CT, USA) with a gradient elution using 0.1% formic acid and acetonitrile is recommended for optimal separation of lipids based on their hydrophobicity [109,110]. Atmospheric pressure chemical ionization in positive mode (450 °C) is used to detect fatty acids and membrane lipids (600–1500 *m*/*z*) [110]. Metabolic flux analysis integrates stable isotope tracing (13C6-glucose for TCA flux, D3-carnitine for β-oxidation) with LC-HRMS (Orbitrap Exploris 480, Thermo Fisher Scientific, Waltham, MA, USA; 240,000 resolution at 200 *m*/*z*, <2 ppm mass error) to quantify isotopologues [89,111]. This approach, combined with Seahorse XF analysis to measure the mitochondrial oxygen consumption rate (OCR), provides a comprehensive assessment of metabolic flux and mitochondrial function [111]. Moreover, samples can also be directly injected into the mass spectrometer without prior separation for mitochondrial primary metabolite detection [112].

### 3.3. Detection Method and Data Analysis

Depending on different experimental needs, metabolomics could be categorized into two types, namely, targeted and non-targeted metabolomics. Targeted metabolomics, often adopted for metabolite quantification via multiple reaction monitoring (MRM), displays high selectivity and sensitivity. On the other hand, non-targeted metabolomics could detect a wide range of metabolites, but it has limitations in quantitative accuracy, dynamic range, sensitivity, and identification accuracy [113].

Currently, developing new methods that integrate the advantages of both targeted and non-targeted metabolomics is an important trend. Pseudo-targeted metabolomics, a new-generation metabolomics approach, involves two main steps for obtaining overall metabolomics data from samples [114]. Firstly, precursor ions and their characteristic ions are obtained from non-targeted metabolomics data collected by GC-MS and LC-MS. Secondly, non-targeted metabolomics data is processed via software packages and then analyzed in the MRM mode. The Xu group and the Zhu group have, respectively, developed MRM-Ion Pair Finder [115] and SWATHtoMRM [116], as systematic and automatic software packages for discovery and characterization of specific MRM ion pairs. Chen et al. have established a mitochondrial metabolite library named MITObolome. Using the selected ion monitoring mode of targeted metabolites, it can identify mitochondrial nucleotides that are difficult to detect. Meanwhile, non-targeted metabolomics detection can identify and discover some molecules outside MITObolome, which is beneficial for maintaining the integrity of mitochondrial DNA [27]. Bayraktar et al. conducted an integrated metabolomics analysis of liver mitochondria, determining the changes in liver mitochondrial metabolites during fasting and re-feeding conditions and their distinct manifestations at the mitochondrial sub-cellular and whole-liver levels [117].

To maximize reproducibility and impact, mitochondrial metabolomics studies should adhere to the FAIR Guiding Principles (Findable, Accessible, Interoperable, Reusable) [118]. Public deposition of raw and processed metabolomics data in dedicated repositories such as MetaboLights [119] or Metabolomics Workbench [120] is strongly recommended. Additionally, compliance with the Metabolomics Standards Initiative (MSI) [121] ensures standardized reporting of experimental metadata, analytical protocols, and data processing workflows, which is critical for cross-study validation and meta-analysis.

### 3.4. Novel Breakthroughs and Applications

Mitochondrial metabolomics studies mitochondrial small molecule dynamics and regulation, central to understanding energy dysfunction, aging, cancer, neurodegenerative disorders (e.g., Alzheimer’s), and metabolic syndrome [92]. Recent advances in single-cell technologies, spatial omics, artificial intelligence, super-resolution imaging, real-time monitoring, and data integration reveal mitochondrial roles in health and disease [122].

Recent innovations in spatial metabolomics, particularly the integration of matrix-assisted laser desorption/ionization mass spectrometry imaging (MALDI-MSI) with cryosectioning or cryo-embedding samples, have enabled the visualization of intramitochondrial metabolite distributions at sub-micrometer resolution [123]. For example, studies have revealed gradients in TCA cycle intermediates across the mitochondrial matrix and cristae structures, providing direct evidence for the role of metabolite compartmentalization in regulating electron transport chain efficiency [124,125,126]. Single-cell mitochondrial metabolomics, exemplified by scMET-seq, utilizes microfluidic chips to isolate mitochondria from individual cells, coupled with targeted LC-MS/MS to detect metabolites such as NAD^+^ and succinate at attomole levels [127]. This approach has been instrumental in revealing tumor heterogeneity, demonstrating that variations in mitochondrial metabolite abundance within a single tumor can predict chemoresistance [128].

Real-time dynamic metabolic monitoring has been revolutionized by the optimization of genetically encoded fluorescent probes, such as iNap, enabling the tracking of ATP, NADH, and reactive oxygen species (ROS) dynamics within mitochondria at millisecond resolution [129,130]. This has challenged traditional steady-state metabolic models, revealing mechanisms of mitochondrial metabolic oscillations and their synchronization with the cell cycle [131]. Furthermore, AI-driven data integration, exemplified by the deep learning model MetaNet, integrates metabolomic data with transcriptomic and proteomic data to predict dynamic regulatory nodes in mitochondrial metabolic pathways [132,133]. This approach has successfully identified novel metabolic biomarkers for complex I deficiency in Parkinson’s disease, such as the abnormal accumulation of 2-hydroxyglutarate [134]. The combination of CRISPR-Cas9 with metabolomics, through the development of CRISPRi metabolomic screening platforms, allows for the systematic analysis of metabolic enzyme functional redundancy networks by targeting and knocking down mitochondria-related genes, followed by metabolite enrichment analysis [135,136]. This has led to the identification of bypass metabolic compensation mechanisms for ETC Complex III [137,138,139].

These technological advancements have driven significant scientific discoveries. In cancer, microfluidic mitochondrial chips have revealed that tumor cells promote immune evasion by secreting succinate, which inhibits mitochondrial function in neighboring immune cells [140,141]. In Alzheimer’s disease models, super-resolution metabolic imaging has revealed a loss of activity of the mitochondrial pyruvate carrier (MPC) around Aβ plaques, leading to impaired acetyl-CoA synthesis [142]. Moreover, mitochondrial metabolites, such as itaconate, have been shown to regulate antioxidant responses through the covalent modification of KEAP1 protein, expanding the non-classical signaling functions of metabolites [143].

Integrating spatial metabolomics with cryo-ET will elucidate metabolite–ultrastructure interactions. Other key directions include developing mitochondrial metabolomic liquid biopsies [144] and applying reinforcement learning to model mitochondrial metabolism for predicting intervention targets [145].

Mitochondrial metabolomics has seen transformative advances in analytical resolution, dynamic monitoring, and multi-omics integration, revealing the role of mitochondrial metabolic plasticity in health and disease. Spatial metabolomics breakthroughs now allow for the study of metabolite gradients within mitochondrial subdomains [126,146]. Such spatial mapping revealed that tricarboxylic acid (TCA) cycle intermediates exhibit distinct partitioning between mitochondrial matrix and cristae compartments, directly modulating electron transport chain efficiency and challenging traditional models of metabolic flux homogeneity [146].

Single-cell mitochondrial metabolomics has emerged as a game-changer in dissecting cellular heterogeneity. The development of scMET-seq (single-cell mitochondrial metabolome sequencing) enabled the ultrasensitive quantification of metabolites like NAD^+^ and succinate at attomolar levels through microfluidic mitochondrial isolation paired with targeted LC-MS/MS [147,148]. This approach revealed unprecedented metabolic diversity within tumors, where differential mitochondrial metabolite abundance among individual cancer cells predicts chemotherapy resistance months before clinical relapse [149]. Concurrently, genetically encoded biosensors such as the iNap series achieved millisecond-resolution tracking of ATP/NADH oscillations in living mitochondria, exposing rhythmic metabolic coupling with cell cycle progression [149,150]. These dynamic measurements overturned static “steady-state” assumptions, revealing mitochondrial metabolism as a pulsatile regulator of cellular timekeeping.

Artificial intelligence has revolutionized data interpretation, with deep learning architectures like MetaNet integrating mitochondrial metabolomics, proteomics, and transcriptomics to predict regulatory nodes in metabolic networks [151,152]. This computational framework identified 2-hydroxyglutarate accumulation as a novel biomarker of complex I deficiency in Parkinson’s disease, linking mitochondrial dysfunction to epigenetic dysregulation. CRISPR-Cas9 functional genomics further accelerated mechanistic discovery—a genome-scale CRISPRi screen mapped metabolic redundancy networks sustaining electron transport chain function, uncovering a compensatory succinate oxidation pathway bypassing complex III inhibition [153].

Clinically, mitochondrial metabolomics has exposed pathogenic mechanisms with therapeutic implications. Microfluidic “mitochondrial chips” demonstrated tumor-derived succinate paracrine signaling that paralyzes immune cell oxidative phosphorylation [154], while super-resolution imaging in Alzheimer’s models revealed Aβ plaque-induced inactivation of mitochondrial pyruvate carriers, disrupting acetyl-CoA synthesis critical for neuronal survival [142,155]. Beyond canonical pathways, metabolites like itaconate were shown to regulate redox homeostasis through covalent KEAP1 modification [156], exemplifying moonlighting roles of mitochondrial intermediates as signaling molecules.

Integration of spatial metabolomics, cryosectioning, and machine learning promises atomic-scale metabolite-structure visualization and prediction of metabolic tipping points. This positions mitochondrial metabolomics as a precision medicine cornerstone, offering dynamic biomarkers and therapeutic targets for metabolic dysregulation.

## 4. Mitochondrial Metabolism and Cancer

**Figure 2:**
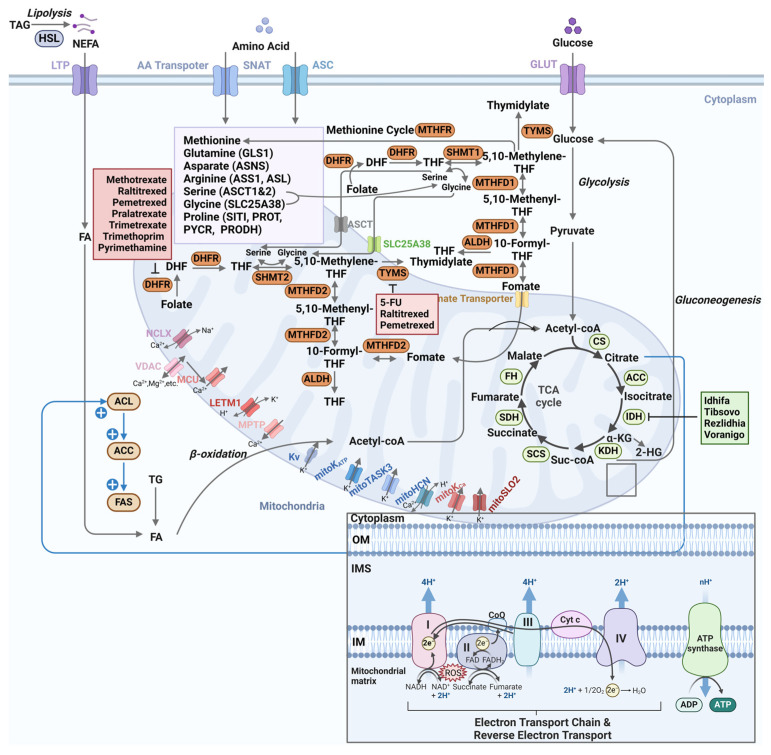
Schematic integration of mitochondrial metabolic networks. This diagram provides a comprehensive overview of the core mitochondrial metabolic pathways and their molecular components, highlighting the synergistic interactions between energy metabolism, redox homeostasis, ion regulation, one-carbon metabolism, fatty acid metabolism, and amino acid utilization. Lipolysis and fatty acid metabolism: Lipolysis, catalyzed by hormone-sensitive lipase (HSL), hydrolyzes triacylglycerols (TAGs) into non-esterified fatty acids (NEFAs) and glycerol, thereby providing substrates for mitochondrial β-oxidation. Fatty acids (FAs) are transported into mitochondria via CD36 and fatty acid-binding proteins (FABPs), where they are activated by acyl-CoA synthetase (ACS) and enter β-oxidation, generating acetyl-CoA, which subsequently feeds into the tricarboxylic acid (TCA) cycle. Amino acid metabolism: Amino acid transporters (e.g., SNAT, ASC, ASCT1/2) mediate the uptake of key amino acids, including glutamine, aspartate, arginine, and serine. Glutamine is converted into α-ketoglutarate (α-KG) by glutaminase (GLS1), which then enters the TCA cycle. The serine/glycine pathway, regulated by serine hydroxymethyltransferase (SHMT) and the folate cycle, provides methyl donors for thymidylate (TYMS/DHFR) synthesis and epigenetic regulation. The methionine cycle, governed by MTHFR, is critical for controlling methylation processes and maintaining redox homeostasis. One-carbon metabolism and nucleic acid synthesis: Folate-mediated one-carbon metabolism generates 5,10-methylene-THF, which is either converted to formate by MTHFD enzymes or utilized in the synthesis of purines and thymidylate. Key enzymes, such as dihydrofolate reductase (DHFR) and thymidylate synthase (TYMS), are therapeutic targets for anticancer agents, including 5-fluorouracil (5-FU) and methotrexate. Ion metabolism and redox regulation: Mitochondrial calcium (Ca^2+^) influx is mediated by the voltage-dependent anion channel (VDAC) and the mitochondrial calcium uniporter (MCU), with efflux facilitated by NCLX and LETM1. Disruption of calcium homeostasis can induce cell death or promote carcinogenesis, as observed in pancreatic and colorectal cancers. Potassium channels (Kv) and the electron transport chain (ETC) regulate reactive oxygen species (ROS) production, influencing tumor progression in malignancies such as leukemia and lung cancer. Reverse electron transport (RET), involving complexes I and III, enhances ROS generation and is implicated in immune metabolic reprogramming. Energy metabolism and targeted therapy: Mutations in key TCA cycle enzymes (e.g., IDH, SDH, FH) lead to the accumulation of oncogenic metabolites (such as 2-HG, succinate, and fumarate), driving tumorigenesis. IDH inhibitors (e.g., Idhifa, Tibsovo) and metabolic enzyme inhibitors (e.g., ACC and FAS inhibitors) have entered clinical trials, providing promising avenues for targeted cancer therapies.

Mitochondrial metabolism is a fundamental driver of tumorigenesis, facilitating bioenergetic, redox, and biosynthetic reprogramming through several critical pathways, including the TCA cycle, ion homeostasis, one-carbon metabolism, fatty acid oxidation, and amino acid utilization. Given the pivotal role of mitochondrial metabolism in these processes, elucidating its contributions to oncogenesis and therapy resistance might help identify specific mitochondrial vulnerabilities that could be strategically targeted in precision anticancer therapies (as shown in Table 3 and Figure 2).

### 4.1. Tricarboxylic Acid Cycle

Mitochondria predominantly rely on the tricarboxylic acid cycle to generate enough energy, which is essential for sustaining cellular fundamental physiological processes [80]. The TCA cycle takes place within the mitochondrial matrix, allowing carbon transfer from glucose, fatty acids, amino acids, and ketone bodies to NADH and FADH2 [73], which provides intermediates for the biosynthesis of signaling molecules determining metabolic rewiring and even the cell fate of cancer cells [157].

Mutation or inhibition of enzymes involved in the TCA cycle has been closely linked to tumor formation. Predominant mutations associated with the accumulation of oncometabolites that promote tumorigenesis are found in three enzymes from the TCA cycle: isocitrate dehydrogenase (IDH), succinate dehydrogenase (SDH), and fumarate hydratase (FH). IDH, a rate-limiting enzyme of TCA cycle, containing three isoforms IDH1, IDH2, and IDH3, could facilitate the conversion of isocitrate to α-KG through oxidative carboxylation [158]. IDH1 and IDH2 mutations frequently exist in multiple human malignancies, including acute myeloid leukemia (AML), glioma, glioblastoma, chondrosarcoma, and cholangiocarcinoma [159,160,161,162]. IDH mutations are gain-of-function mutations [163], turning α-KG into oncometabolite 2-HG that initiates cellular redox imbalance and tumorigenesis [163,164]. SDH, also regarded as ETC Complex II, is the only ETC Complex functioning in both the TCA cycle and OXPHOS, oxidizing succinate to fumarate. The defects or mutations of SDH have been disclosed in an extensive range of human tumors, including paraganglioma, pheochromocytoma, gastrointestinal stromal tumors, renal cell cancer (RCC), thyroid tumors, seminomatous testicular cancer, and neuroblastic tumors [165,166,167,168,169,170,171]. FH converts fumarate to malate. Mutations in the FH gene have been implicated in the development of renal cell carcinoma (RCC) [172], as well as in ovarian and Leydig cell cancers [173]. Mutations of SDH and FH (both tumor suppressors) are loss-of-function mutations and cause abnormal accumulation of succinate and fumarate, which promotes tumor growth and development [174]. Loss-of-function mutations in tumor suppressors such as FH or SDH are challenging to target, while targeting gain-of-function mutations such as those found in IDH is much more feasible. To date, four approved IDH inhibitors have emerged, including Idhifa [175], Tibsovo [176], Rezlidhia [177], and Voranigo [178]. Pharmacometabolomics studies using LC-MS demonstrated that enasidenib treatment reduces 2-HG levels by 90% in patient serum, correlating with restored ten-eleven translocation (TET) enzyme activity and DNA demethylation [179,180]. MRM-based monitoring further validated the 2-HG/glutamic acid ratio as a predictive biomarker for IDH-1 mutation status in gliomas [181]. Idhifa (enasidenib) is the first approved IDH2 mutation-targeted oral inhibitor for the treatment of relapsed or refractory (R/R) AML [182]. Tibsovo (Ivosidenib) is an FDA-approved therapy for the treatment of cholangiocarcinoma and R/R AML patients with IDH1 mutation [183,184]. Rezlidhia (olutasidenib) is another novel mutant IDH1 inhibitor for the treatment of R/R AML [185]. Voranigo (vorasidenib) is the first FDA-approved IDH1 and IDH2 inhibitor for the treatment of grade 2 IDH-mutant glioma [178]. Therefore, the intimate relationship between enzyme alterations in the TCA cycle and tumor initiation has been highlighted, which points toward the development of inhibitors targeting the TCA cycle as a potential therapy for cancer patients.

### 4.2. Redox Homeostasis

Maintaining mitochondrial redox homeostasis, defined as the equilibrium of the intracellular redox state, is crucial for cellular survival and functionality, which hinges on two critical pathways: the electron transport chain (ETC) and reverse electron transport (RET) [186].

The ETC serves as a pivotal element in intracellular bioenergetics, facilitating ATP synthesis via OXPHOS. In the OXPHOS process, electrons originating from donors such as NADH and FADH2 are transferred to oxygen, potentially producing mtROS during incomplete reduction [73]. Notably, ROS generation predominantly occurs at complex I and complex III of the ETC, where electron leakage facilitates the formation of O_2_^•−^ [73,187]. Although moderate ROS levels are essential for cellular signal transduction and adaptation, excessive ROS could induce oxidative stress, damaging intracellular biomolecules such as DNA, proteins, and lipids [188,189,190].

RET describes the phenomenon where electrons flow from complex II to complex I, typically under specific metabolic conditions such as fatty acid oxidation, inducing changes in the NADH/FADH2 ratio and over-reduction in the coenzyme Q pool [191]. Additionally, these RET-derived ROS play crucial roles in modulating cellular metabolism and immune responses, which regulate the cytokine production of macrophages [192] and adjust the availability of metabolic substrate production from the ETC pathway [193].

Mitochondrial redox homeostasis is crucial for maintaining cellular function under normal physiological conditions. However, pathological conditions like ischemia–reperfusion injury, inflammation, and tumorigenesis can disrupt this balance, leading to excessive ROS production and oxidative stress. Elucidating the regulatory mechanisms of mitochondrial redox homeostasis is essential for understanding and treating these diseases [194].

Recent studies have shown that ROS play a significant role in myocardial ischemia–reperfusion injury (MIRI), contributing to mitochondrial dysfunction and lipid peroxidation [195]. Lipidomic profiling using MALDI-TOF imaging mass spectrometry revealed an increase in peroxidized cardiolipin CL 72:8-OOH in myocardial ischemia–reperfusion injury (MIRI) models, indicative of mitochondrial oxidative damage. Consistent with this, LC-MS/MS analyses detected elevated levels of 4-hydroxynonenal (4-HNE) protein adducts—a well-established biomarker of lipid peroxidation—further confirming oxidative stress activation [196,197]. Analogous redox dysregulation patterns were observed in ROS1 fusion-driven non-small cell lung cancer (NSCLC) [198]. Targeted metabolomics in these tumors identified succinate accumulation as a key metabolic shift, which promotes RET-dependent signaling via reactive oxygen species (ROS) generation, illustrating a ROS1 fusion-dependent mechanistic pathway [199,200]. Similarly, research on non-small cell lung cancer (NSCLC) has identified rearrangements in ROS1 and RET genes as novel driver mutations, offering new therapeutic targets [201,202]. HILIC-MS analysis in NSCLC tissues demonstrated a significant reduction in the NAD^+^/NADH ratio (Table 1), indicating impaired OXPHOS and enhanced glycolysis [203,204]. Concurrently, redox metabolomics revealed a 50% reduction in glutathione (GSH) and increased oxidized glutathione (GSSG), linking mtROS overproduction to cisplatin resistance [205,206].

Overall, the study of mitochondrial redox homeostasis is a dynamic field with ongoing research continuously revealing its significance in both physiological and pathological processes and its potential as a therapeutic target.

### 4.3. Ion Metabolism

The mitochondrial membranes, both the IM and OM, are equipped with numerous ion channels and transporters, working together to sustain cellular ion equilibrium, vital in regulating cell integrity under physiological and pathological conditions [207,208]. Ion channels and transporters, especially those localized in the IM, are indispensable for intracellular communication between mitochondria and the cytoplasm [209].

Mitochondria integrates cell metabolism with calcium (Ca^2+^) transportation, by regulating not just mitochondrial Ca^2+^ concentration but also the comprehensive cellular Ca^2+^ signaling network [210,211]. The mitochondrial Ca^2+^ import is dependent on the voltage-dependent anion channel (VDAC) and the mitochondrial calcium uniporter (MCU), particularly the MCU. The VDAC is key for Ca^2+^ shuttling between mitochondria and cytoplasm, whereas the MCU is primarily responsible for controlling intramitochondrial Ca^2+^ [212,213]. Mitochondrial Ca^2+^ efflux is predominantly dependent on the Na^+^/Ca^2+^ exchanger (NCLX), Ca^2+^/H^+^ exchanger (LETM1), and MPTP [214]. Disruption of the balance between calcium influx and efflux could induce various diseases [215,216,217]. MCU overexpression would activate Keap1-Nrf2 by elevating mtROS, which is pivotal for metastasis and poor prognosis of pancreatic ductal adenocarcinoma (PDAC) [218]. Moreover, MCU overexpression could facilitate colorectal cancer (CRC) progression by increasing mitochondrial Ca^2+^ import through the ROS/NF-κB signaling pathway [218]. Ion-coupled metabolomics in PDAC revealed that MCU overexpression significantly elevates mitochondrial Ca^2+^ [219], detected via Rhod-2 AM fluorescence imaging, which correlates with an increase in citrate and activation of the Keap1-Nrf2 antioxidant pathway [220]. In CRC models, calcium overload promotes ROS accumulation through mitochondrial reverse electron transport and/or NOX activation [221]. LC-MS-based redox metabolomics revealed a concomitant decrease in the NADPH pool of tumors, reflecting compromised antioxidant capacity [222]. Mitochondrial Ca^2+^ dysregulation could induce cell death. However, the upregulation of MUC20 variant 2 inhibits Ca^2+^ accumulation within mitochondria, promoting cell survival and instigating chemotherapy resistance in gastric cancer [223]. Consequently, targeting the MUC (whose overexpression and mutation are highly correlated with tumorigenesis) and regulating mitochondrial Ca^2+^ metabolism might be promising strategies for cancer treatment.

Mitochondrial potassium (K^+^) balance assists in preserving mitochondrial size, which is mediated by Ca^2+^-activated K^+^ (K_Ca_), voltage-gated K^+^ (K_V_), mitoK, mitoTASK3, mitoSLO2, and mitoHCN channels. MitoKv1.3 is abnormally active and upregulated in primary leukemic B cells during chronic lymphocytic leukemia (CLL) [224]. Additionally, inhibition of mitoKv1.3 via PAPTP could ameliorate CLL by increasing mtROS, releasing cytochrome C, and inducing cell death [225]. K_Ca_3.1 overexpression could promote the pathogenesis of non-small cell lung cancer (NSCLC), while inhibition of K_Ca_3.1 could suppress NSCLC via increasing ROS production and mitigating erlotinib resistance [226,227]. In short, targeting K^+^ channels in mitochondria for cancer therapy has gained significant attention due to their activation in many cancers. Although the development of ion channel-targeted drugs has gained great advances in nervous system disorders, research for cancer treatment still needs further exploration.

### 4.4. One Carbon Metabolism

One-carbon metabolism, encompassing a network of interconnected cytosolic and mitochondrial reactions, involves the generation, transfer, and utilization of one-carbon units. These units, primarily derived from the folate cycle and methionine cycle, are essential for the biosynthesis of thymidine, methionine, serine/glycine, and purine [228]. The methionine cycle often takes place in the cytosol for methionine production, while the folate cycle occurs in the cytosol and mitochondria for biosynthesis of serine/glycine, thymidine, and purine [229].

One-carbon metabolism is crucial for DNA synthesis, methylation reactions, and amino acid metabolism. This process involves several key enzymes, including serine hydroxymethyltransferase (SHMT), methylenetetrahydrofolate dehydrogenase (MTHFD), aldehyde dehydrogenase (ALDH), thymidylate synthase (TYMS), dihydrofolate reductase (DHFR), and DNA methyltransferase (DNMT) [230]. SHMT, an enzyme in the folate cycle, catalyzes the interconversion of serine and glycine while generating one-carbon units essential for purine and thymine synthesis [231]. Similarly, MTHFD significantly contributes to folate metabolism by catalyzing the dehydrogenation of methylenetetrahydrofolate, which is vital for the synthesis of purine and thymine [232]. Furthermore, ALDH primarily functions in ethanol metabolism by oxidizing acetaldehyde to acetic acid, and it also participates in the metabolism of various aldehyde compounds, thereby protecting cells from aldehyde toxicity [233]. Additionally, TYMS catalyzes the synthesis of thymidylate, which is critical for DNA synthesis and is closely associated with the prognosis of several cancers [234]. Moreover, DHFR catalyzes the reduction of dihydrofolate to tetrahydrofolate, the pivotal step in folate metabolism; thus, inhibiting DHFR can lead to ‘thymineless death’, making it a significant target for anticancer therapies [235]. Finally, DNMT catalyzes DNA methylation reactions, an important epigenetic regulatory mechanism that is closely linked to the onset and progression of various diseases, particularly tumors [236].

Overexpression of folate cycle enzymes, including SHMT and MTHFD, has been the most significantly overexpressed metabolic genes in an analysis of nineteen different cancer types [237]. Furthermore, SHMT2, MTHFD2, and MTHFD1L could accelerate the proliferation of NCI-60 cancer cells [238]. Increased MTHFD2 levels are also associated with the migration, invasion, and poor clinical prognosis of breast cancer [239], NSCLC [240], RCC [241], hepatocellular carcinoma (HCC) [242], squamous cell carcinoma (SCC) [243], and ovarian cancer [244]. Upregulation of SHMT2, MTHFD2, and aldehyde dehydrogenase 1L2 (ALDH1L2) is linked to CRC pathogenesis [245]. Pseudotargeted metabolomics (SWATHtoMRM) identified a 3-fold enrichment of 5,10-methylene-THF in breast cancer, with GC-MS confirming formate accumulation as a predictor of poor prognosis [246]. Folate cycle activation also increased dTMP synthesis (quantified by ^13^C-glucose tracing), driving 5-FU resistance in CRC and breast carcinoma [247,248].

One-carbon metabolism enzymes are essential for normal cellular activities, and their dysregulation is highly associated with tumorigenesis [230]. Elucidating the mechanisms of these one- carbon metabolism enzymes could explore their potential as therapeutic targets. Currently, the pharmaceutical landscape targeting one-carbon metabolism primarily encompasses TYMS inhibitors such as 5-fluorouracil (5-FU), Raltitrexed, and Pemetrexed [249]; DHFR inhibitors including Methotrexate, Raltitrexed, Pemetrexed, Pralatrexate, Trimetrexate, Trimethoprim, and Pyrimethamine [250]; and DNMT1 inhibitors like Azacytidine and Decitabine, etc. [251].

### 4.5. Fatty Acid Metabolism

Fatty acids are amphipathic molecules essential for energy metabolism. Mitochondrial fatty acid metabolism is a complex and highly regulated network essential for maintaining energy homeostasis, lipid storage, and overall cellular function [252]. The mitochondrial uptake of fatty acids is facilitated by specific transport proteins such as fatty acid-binding proteins (FABPs) and plasma membrane transporters like CD36 [253,254]. Lipolysis, catalyzed by hormone-sensitive lipase (HSL), breaks down triglycerides into free fatty acids and glycerol, providing substrates for mitochondrial metabolism [255] [256]. De novo fatty acid synthesis begins with acetyl-CoA and involves multiple enzymatic steps catalyzed by fatty acid synthase (FAS) [257]. Before entering the mitochondria, fatty acids undergo activation by acyl-CoA synthetase to form acyl-CoA [258]. Peroxisomal fatty acid β-oxidation (FAO) complements mitochondrial FAO, particularly for very-long-chain fatty acids (VLCFAs) [259]. Lipidomics via LC-MS in HSL-high tumors showed a 4-fold increase in palmitate and oleate levels [260], while MALDI-TOF imaging revealed spatial accumulation of acylcarnitines (C16:1, C18:1) in mitochondrial subregions [261]. These FAO intermediates correlated with ATP overproduction (measured by Seahorse assay) and metastasis in xenograft models [260,262].

CD36 mediates the uptake of long-chain fatty acids from the extracellular environment into the cytoplasm [263]. Cytosolic fatty acid-binding proteins (FABPs) then bind to these fatty acids, facilitating their movement towards the mitochondria [264]. The regulation of CD36-mediated fatty acid uptake and its implications in lipid-related diseases have been extensively studied [265,266,267,268]. Recent studies demonstrate that elevated CD36 expression in various tumors fosters lipid internalization, promoting metabolic reprogramming toward heightened proliferation and metastatic capacity—an emerging hallmark of cancer progression [269,270].

Hormone-sensitive lipase (HSL) plays a central role in lipolysis within adipocytes [255]. Hormonal signals such as adrenaline or glucagon activate HSL through phosphorylation cascades, leading to the breakdown of triglycerides into free fatty acids and glycerol [271]. Currently, aberrant HSL activity in the tumor microenvironment has been linked to lipid supply for cancer cell growth, suggesting that targeting lipolysis may interfere with the metabolic adaptability of tumors and impede their progression [272,273].

The process of fatty acid synthesis occurs primarily in the cytoplasm, starting with acetyl-CoA carboxylated to malonyl-CoA by acetyl-CoA carboxylase (ACC), the rate-limiting step [274]. Fatty acid synthase (FAS) then elongates the acyl chain. The newly synthesized fatty acids can be stored as triglycerides or modified for transport to various cellular compartments [275]. In tumor cells, upregulated ACC and FAS catalyze de novo lipogenesis to fulfill heightened demands for membrane biosynthesis and signaling lipids, driving oncogenesis and conferring growth advantages; indeed, inhibitors of these enzymes have demonstrated anticancer potential in preclinical models [276,277,278,279].

Fatty acids must be activated by acyl-CoA synthetase in the cytoplasm to form acyl-CoA. This high-energy reaction is essential for subsequent metabolic steps such as transport into the mitochondria and β-oxidation [280]. Elevated expression of acyl-CoA synthetases in multiple cancer types correlates with advanced tumor grades and poor patient prognosis, underscoring these enzymes as potential metabolic checkpoints for therapeutic intervention [281,282].

Peroxisomal fatty acid β-oxidation (FAO) is vital for the oxidation of very-long-chain fatty acids (VLCFAs). The process begins with acyl-CoA oxidase, followed by enoyl-CoA hydratase, 3-hydroxyacyl-CoA dehydrogenase, and thiolase. Disorders in peroxisomal FAO can lead to severe metabolic consequences [283]. Emerging evidence indicates that peroxisomal FAO contributes significantly to cancer cell fitness, particularly via VLCFA catabolism that supports membrane remodeling and redox balance; its dysregulation can promote tumorigenesis and metastasis, making it a potential therapeutic target [284,285].

Mitochondrial fatty acid metabolism involves a series of intricately coordinated processes essential for cellular function and energy homeostasis [13]. Understanding these processes at a molecular level is crucial for research in metabolic disorders, obesity, and diabetes. Future studies should focus on the regulatory mechanisms and potential therapeutic targets within this network to address metabolic dysfunctions.

### 4.6. Amino Acid Metabolism

Tumor cells exhibit a high demand for energy to support their proliferative activities [286,287]. Amino acid metabolism, beyond protein synthesis, plays a crucial role in meeting this energy requirement and can even determine the fate of tumor cells [288,289]. Non-essential amino acids (such as glutamine, aspartate, arginine, serine, glycine, and proline) are often synthesized within the tumor cells [290], whereas essential amino acids (such as methionine) are typically acquired from external sources [291]. The subsequent section delves into the metabolic pathways and implications of pivotal amino acids in terms of tumor biology and investigates potential therapeutic strategies targeting these pathways.

Glutamine, though classified as a non-essential amino acid, is critical for maintaining mitochondrial oxidative metabolism in certain tumor cells, due to its consumption far exceeding cellular synthesis rates [292]. Glutamine enters tumor cells primarily through amino acid antiporters such as ASCs (Na^+^-dependent alanine-serine-cysteine transporters ASCT1 and ASCT2) and SNATs (Na^+^-coupled neutral amino acid transporters, including SNAT1, SNAT2, and SNAT5) [293,294]. Additionally, it can be transported via broadly specific amino acid transporters like sodium-coupled neutral and basic amino acid transporter B(0+) (ATB0+, SLC6A14) and the more selective amino acid transporter solute carrier family 38 member 5 (SLC38A5) [295]. Glutaminase 1 (GLS1), the first and rate-limiting enzyme in glutamine catabolism, hydrolyzes glutamine to glutamate and ammonia [296]. ^13^C-glutamine tracing combined with HILIC-MS in NSCLC demonstrated that GLS1 overexpression increases intracellular glutamate by 5-fold and α-KG by 3-fold, supporting TCA cycle anaplerosis [297]. Untargeted metabolomics further linked glutamine-derived proline (detected by GC-MS) to collagen remodeling and immune evasion [298]. Glutamate is then converted to α-KG, which enters the TCA cycle for complete oxidation, producing substantial energy critical for maintaining mitochondrial energy metabolism in tumor cells [299]. GLS1 expression is upregulated in various cancer types, including HCC, breast cancer, melanoma, lung cancer, and RCC [297,300,301,302,303,304]. Inhibition of GLS1 blocks tumor cell growth by reducing glutamine levels and decreasing mitochondrial ATP and NADH production [305].

In the absence of glutamine, tumor cells display adaptive metabolic reprogramming through the exogenous uptake of aspartate [306]. Aspartate mainly enters tumor cells via transporters. Endogenous aspartate production in tumor cells largely depends on the synthesis by asparagine synthetase (ASNS) [307]. To date, the regulatory role of aspartate in tumor control remains unclear. ASNS gene expression is observed to be reduced in some acute lymphoblastic leukemia subtypes [308] but overexpressed in other tumor types such as NSCLC, colorectal cancer CRC, and breast cancer [309,310,311], promoting tumor growth and metastasis via mTORC1 activation, enhanced stress response, epithelial–mesenchymal transition, and cell cycle progression, etc. [309,310,311]. Aspartate can influence mitochondrial respiration and ATP synthesis in tumor cells by modulating mTORC1 activity and upregulating activating transcription factor 4 (ATF4), altering tumor cell activity [312].

Arginine is essential for nitric oxide and polyamine synthesis, with its intracellular production predominantly dependent on the activity of arginine succinate synthase 1 (ASS1) and arginine succinate lyase (ASL) [313]. The expression of ASS1 is inhibited in many tumor cells, necessitating an external supply of arginine via cationic amino acid transporters and heteromeric amino acid transporters [314,315]. Studies have shown that reducing dietary arginine intake can induce mitochondrial dysfunction and inhibit oxidative phosphorylation (OXPHOS), leading to tumor cell apoptosis [316].

Serine is an important non-essential amino acid for tumor tissues, capable of being both exogenously acquired and synthesized via glycolysis [317]. Tumor cells primarily uptake extracellular serine through ASCT1 and ASCT2 transporters [318,319]. Recent studies highlight serine as a crucial regulator of cellular redox balance, emphasizing its indispensable role in glutathione biosynthesis where it furnishes vital reducing equivalents [320]. This function is particularly significant as it upholds the redox homeostasis that facilitates tumor cell proliferation. Of therapeutic relevance, interventions such as serine deprivation and nutritional starvation have demonstrated efficacy in decelerating tumor growth and enhancing survival rates in vivo [321,322], thereby emphasizing the potential of targeting serine metabolism as a novel anticancer strategy. Intracellularly, serine could be converted to glycine by demethylation, liberating a methyl group that subsequently integrates into the folate cycle [323,324], which would further induce cancer immune evasion through PD-L1 upregulation [325].

Intracellular proline accumulation occurs through both exogenous uptake and metabolic generation [326]. This process is primarily facilitated by the broadly specific sodium/symporter 1 (SIT1) and the brain-specific Na^+^-dependent proline transporter PROT (SLC6A7) [327,328]. Metabolically, proline is mainly generated from extracellular matrix collagen catabolism and pyrroline-5-carboxylate reductase (PYCR)-catalyzed synthesis from glutamate [329]. PYCR activity correlates with increased protein synthesis and cancer growth. Notably, high expression of both PYCR and proline dehydrogenase (PRODH), which catalyzes the oxidation of proline, is linked to enhanced mitochondrial OXPHOS, producing more ATP involved in various cancer progressions [330]. Future research should aim at elucidating the molecular mechanisms underlying proline metabolism in tumor cells to develop more effective and specific treatments.

Methionine is an essential amino acid indispensable for regulating methylation reactions in the body. Through its coupling with the folate metabolic pathway, methionine plays a critical role in maintaining cellular redox status and promoting tumor cell survival via the epigenetic modification of oncogenes and tumor suppressors [331]. Although tumor cells retain the ability to endogenously synthesize methionine, this process is insufficient to sustain their accelerated growth and proliferative needs [332]. Compelling evidence underscores that the progression of numerous tumorigenic processes is predominantly fueled by the exogenous acquisition of methionine, a phenomenon termed the “Hoffman effect” [333]. Enhanced methionine uptake, found in leukemia, breast cancer, HCC, and neuroblastoma [334,335], is a key metabolic alteration that promotes tumorigenesis. ChIP-seq integrated with LC-MS metabolomics in cancer cachexia revealed that methionine restriction reduces the SAM/SAH ratio by 70%, leading to global DNA hypomethylation [298]. MRM-MS quantified methionine cycle intermediates as biomarkers of PD-L1 upregulation and immunotherapy failure [336,337]. Methionine plays essential roles in regulating nucleic acid and chromatin methylation, modulating intracellular redox balance, and driving rapid tumor cell proliferation [338]. These findings regard methionine as a critical factor in cancer metabolism, highlighting its potential as a therapeutic target.

Targeting amino acid metabolism primarily involves developing inhibitors of amino acid transporters and designing amino acid deprivation strategies [289,339]. However, the redundancy of amino acid transporters, which can recognize and transport multiple amino acids, along with potential reverse transport, complicates the precise regulation of cellular amino acid metabolism [340]. Additionally, amino acid deprivation therapies often lead to adaptive resistance in tumor cells, further hindering therapeutic development [341]. To advance this field, future research should focus on elucidating the molecular mechanisms underlying amino acid transport and metabolism in tumor cells, paving the way for more effective and specific treatments.

**Table 3:** Mitochondrial metabolic reprogramming in oncogenesis.

Cancer Type	Metabolite	Change	Association	Refs.
AML	2-HG	Accumulation	IDH1/IDH2 mutations: 2-HG accumulation inhibits TET/HDMs, causing DNA hypermethylation and differentiation block; promotes redox imbalance and leukemogenesis.	[83,99,100,101,102,114,115,116,117,118,119,120,121]
	Glutamine	Increased	GLS1 upregulation supports TCA cycle anaplerosis via α-KG.	[213,214,215,216,217,218,219,220,221]
Glioma	2-HG	Accumulation	IDH1 mutations: Chromatin remodeling impairs differentiation, driving oncogenesis via epigenetic dysregulation.	[83,98,99,117]
	NAD^+^/NADH Ratio	Decreased	Enhanced glycolysis and suppressed OXPHOS.	[73,123]
Cholangiocarcinoma	2-HG	Accumulation	IDH1 mutations: Epigenetic dysregulation drives chemoresistance and tumor progression.	[101,120]
	Fatty Acid	Upregulated	CD36—mediated lipid uptake fuels energy production and metastasis.	[186,187]
Paraganglioma	Succinate	Accumulation	SDHB/SDHD mutations: HIF-1α stabilization activates VEGF, promoting angiogenesis.	[104,105,106]
	ROS	Increased	Succinate accumulation inhibits prolyl hydroxylases, enhancing HIF-1α-mediated survival.	[104,127]
RCC	Fumarate	Accumulation	FH mutations: DNA alkylation and NRF2 activation promote antioxidant defense; fumarate accumulation induces DNA damage and tumorigenesis.	[111,164]
	Glutathione	Increased	Enhanced GSH synthesis compensates for oxidative stress from fumarate accumulation.	[124,126]
NSCLC	Glutamine	Increased uptake	GLS1 overexpression fuels α-KG production for TCA cycle and nucleotide synthesis, supporting proliferation.	[149,150,219,222]
	Aspartate	Increased	ASNS upregulation supports mTORC1-driven proliferation and metastasis.	[225,226,227]
HCC	Glutamine	Increased uptake	Glutaminolysis supports ATP production and redox balance via GSH synthesis.	[215,251]
	Acetyl-CoA	Accumulation	FASN overexpression drives de novo lipogenesis for membrane biosynthesis.	[192,193,194]
Breast Cancer	Serine/Glycine	Increased synthesis	SHMT2/MTHFD2 overexpression supports nucleotide synthesis and redox homeostasis.	[160,162]
	Fatty Acid	Upregulated	ACC/FASN upregulation provides lipids for rapid proliferation.	[191,192,193]
CRC	Aspartate	Increased uptake	ASNS-mediated aspartate synthesis supports mTORC1 activation and EMT, promoting metastasis.	[225,226,227]
	Butyrate	Decreased	Dysbiosis reduces butyrate levels, impairing colonocyte metabolism and promoting inflammation.	[179,202]
PDAC	Mitochondrial Ca^2+^	Increased influx	MCU overexpression activates ROS/NF-κB signaling, driving metastasis.	[145]
	Ketone Bodies	Increased	β-Hydroxybutyrate: Alternative energy source under hypoxia.	[81,172]
CLL	Mitochondrial K^+^	Dysregulated flux	MitoKv1.3 upregulation inhibits apoptosis, promoting survival.	[147,148]
	ATP/ADP Ratio	Decreased	OXPHOS suppression shifts energy reliance to glycolysis.	[74,145]
Ovarian Cancer	Folate Cycle Intermediates	Increased	MTHFD2 overexpression supports purine synthesis and chemoresistance.	[167]
	Proline	Accumulation	PYCR1-driven proline synthesis supports redox balance and tumor growth.	[245,246]
Melanoma	Glutamine	Increased uptake	GLS1 inhibition reduces α-KG levels, suppressing TCA cycle and proliferation.	[218]
	Lactate	Accumulation	Warburg effect: Dominant glycolysis with suppressed mitochondrial respiration.	[22,81]

## 5. Conclusions

In conclusion, this review highlights the transformative impact of mitochondrial metabolomics in cancer research, elucidating the intricate interplay between mitochondrial function and tumor metabolism. Our findings highlight the significance of mitochondrial metabolites not only as biomarkers for cancer progression but also as potential therapeutic targets. Despite the challenges associated with high-purity mitochondrial sample isolation and low-abundance metabolite detection, advancements in mass spectrometry techniques offer promising avenues for overcoming these obstacles.

Future progress in mitochondrial metabolomics will depend on collaborative data sharing. We advocate for community adoption of the FAIR principles and mandatory data deposition in public repositories. Standardization via the Metabolomics Standards Initiative will further enhance cross-platform comparability and accelerate therapeutic discovery.

Future research should prioritize the identification of specific mitochondrial metabolites and their pathways in tumorigenesis, with an emphasis on developing targeted therapeutic interventions. Additionally, integrating novel methodologies, such as nanotechnology and advanced imaging techniques, will enhance our understanding of mitochondrial dynamics in cancer cells. Ultimately, insights gained from mitochondrial metabolomics could pave the way for innovative diagnostic and therapeutic strategies, fostering a new era of precision medicine in oncology.

## Figures and Tables

**Table 2:** Methods of mass spectrometry-based mitochondrial metabolomics in cancer research.

Metabolite Class	Sample Preparation	Chromatography	Ionization	Key Applications	Ref.
TCA intermediates	Methanol/water (8:2)	HILIC (BEH Amide)	ESI(−)	Redox ratio (NAD^+^/NADH)	[82]
Amino acids	Methanol/water (9:1)	GC (DB-5MS)	EI	Isotopomer flux (U-13C-glutamine)	[91]
Cardiolipins	Chloroform/methanol (2:1)	RP-C18	APCI(+)	Cristae membrane dynamics	[109]
Acylcarnitines	Acetonitrile precipitation	HILIC (ZIC-HILIC)	ESI(+)	β-oxidation disorders	[104]
Nucleotides	Perchloric acid (0.3 M)	Ion-pairing RP (C18)	ESI(−)	ATP/ADP energy charge	[106]

## Data Availability

Not applicable.

## Abbreviations

The following abbreviations are used in this manuscript:

Abbreviation	Full Name
mtDNA	Mitochondrial DNA
OM	Outer Membrane
IMS	Intermembrane Space
MPTP	Mitochondrial Permeability Transition Pore
IM	Inner Membrane
ETC	Electron Transport Chain
OXPHOS	Oxidative Phosphorylation
NADH	Nicotinamide Adenine Dinucleotide
FADH2	Flavine Adenine Dinucleotide
α-KG	Alpha-Ketoglutarate
2-HG	2-Hydroxyglutarate
IDH	Isocitrate Dehydrogenase
SDH	Succinate Dehydrogenase
FH	Fumarate Hydratase
TET	Ten-Eleven Translocation
FAO	Fatty Acid Oxidation
GLS1	Glutaminase 1
ASCT	Alanine–Serine–Cysteine Transporter
SNAT	Sodium-Coupled Neutral Amino Acid Transporter
SOD	Superoxide Dismutase
CAT	Catalase
GPx	Glutathione Peroxidase
HSL	Hormone-Sensitive Lipase
FAS	Fatty Acid Synthase
ACC	Acetyl-CoA Carboxylase
DHFR	Dihydrofolate Reductase
TYMS	Thymidylate Synthase
ALDH	Aldehyde Dehydrogenase
SHMT	Serine Hydroxymethyltransferase
MTHFD	Methylenetetrahydrofolate Dehydrogenase
DNMT	DNA Methyltransferase
KCa	Calcium-Activated Potassium Channel
KV	Voltage-Gated Potassium Channel
VDAC	Voltage-Dependent Anion Channel
MCU	Mitochondrial Calcium Uniporter
NCLX	Sodium/Calcium Exchanger
LETM1	Calcium/Hydrogen Exchanger
FFE	Free-Flow Electrophoresis
CE	Capillary Electrophoresis
FFF	Field-Flow Fractionation
FAOS	Fluorescence-Activated Organelle Sorting
AP	Affinity Purification
LCM	Laser Capture Microdissection
OTs	Optical Tweezers
FluidFM	Fluid-Force Microscopy
OCR	Oxygen Consumption Rate
ECAR	Extracellular Acidification Rate
mtROS	Mitochondrial Reactive Oxygen Species
PDAC	Pancreatic Ductal Adenocarcinoma
NSCLC	Non-Small Cell Lung Cancer
RCC	Renal Cell Carcinoma
HCC	Hepatocellular Carcinoma
SCC	Squamous Cell Carcinoma
AML	Acute Myeloid Leukemia
TEM	Transmission Electron Microscope
